# High Seroprevalence of Dengue Virus Infection in Sudan: Systematic Review and Meta-Analysis

**DOI:** 10.3390/tropicalmed5030120

**Published:** 2020-07-18

**Authors:** Adel Hussein Elduma, A. Desiree LaBeaud, Jessica A. Plante, Kenneth S. Plante, Ayman Ahmed

**Affiliations:** 1 National Public Health Laboratory, Federal Ministry of Health, Khartoum 11111, Sudan; dumanet@gmail.com; 2 School of Medicine, Stanford University, Stanford, CA 94305, USA; dlabeaud@stanford.edu; 3 World Reference Center for Emerging Viruses and Arboviruses, University of Texas Medical Branch, Galveston, TX 77555, USA; japlante@utmb.edu (J.A.P.); ksplante@utmb.edu (K.S.P.); 4 Department of Microbiology and Immunology, Institute for Human Infections and Immunity, University of Texas Medical Branch, Galveston, TX 77555, USA; 5 Institute of Endemic Diseases, University of Khartoum, Khartoum 11111, Sudan

**Keywords:** dengue fever, dengue virus, systematic reviews, meta-analysis, emergence, re-emergence, arboviruses, Sudan

## Abstract

The goal of this study was to systematically review the published data on dengue virus (DENV) seroprevalence in Sudan and to estimate disease burden through meta-analysis. We searched, reviewed, and extracted online available reports on DENV in Sudan. Among 168 identified records, 19 were selected. Dengue infections were documented in 11/18 states. The overall seroprevalence of DENV in Sudan was estimated to be 27%, while the prevalence of dengue IgM was 22% and IgG was 38%. The prevalence of dengue estimated from community and hospital-based cross-sectional studies were 26% and 30% respectively. Additionally, one cohort study and a single PCR-based study reported a prevalence of 1% and 4%, respectively. Regional analysis revealed that the variation in seroprevalence in East, North, West, and Central Sudan was 23%, 24%, 36% and 43%, respectively. Interestingly, we found that DENV is circulating countrywide with a significant spatiotemporal variation in the disease seroprevalence. Furthermore, publications on dengue prevalence are temporally and geographically fragmented, perhaps due to limited resources. However, this gap in data and knowledge highlights the urgent need for a country-wide surveillance system and continued study of dengue burden in Sudan to accurately estimate the disease prevalence and determine the associated risk factors.

## 1. Introduction

Dengue fever (DF) is an arthropod-borne viral disease (arbovirus) caused by dengue virus (DENV) which circulates in tropical and sub-tropical areas where the environment is suitable for vector breeding [1]. Humans are the main carrier of the virus and the amplifying host with non-human primates plays a considerable role in sylvatic cycle [1,2]. Dengue virus is mainly transmitted by a day biting mosquito, *Aedes aegypti*, which is a container breeder that thrives in urban environments [3,4]. The virus has four closely related serotypes (DENV 1–4) [5]. DENV infection ranges from severe disease, which can present with hemorrhage and shock, to sub-clinical asymptomatic infection, and it is commonly under-recognized in children [6,7]. Dengue is endemic in more than 128 countries worldwide, with half of the world population at risk of the disease [5]. Global dengue incidence has increased dramatically and accounted for 390 million new infections per year of which 96 million have developed apparent disease [5].

Several factors are associated with the recent resurgence of DENV, including increases in urbanization, international travel, trade, and lack of effective vector control [5,7]. Infection with DENV has been described in the Sudan as early as 1906 in the eastern part of the country [8], with frequent epidemics confined to that region [8,9,10]. DENV outbreaks were later associated with the flooding in Khartoum and the Northern state in 1988 and 1989, respectively [11,12]. DENV has recently emerged in the southern and western regions of Sudan, causing large epidemics in refugee camps over the last 6 years [13,14,15]. These recent epidemics of DENV have followed drastic changes in physical, social, and environmental factors as a result of the war and humanitarian crisis in these regions [13,14,16]. As a result, DENV has become a serious public health issue in different areas of Sudan in recent years [13,14].

Investigations into DENV incidence in Sudan have produced variable results, presumably due to geographic, temporal, and methodological differences [17]. Even DENV-endemic areas are witnessing changes in the virus serotypes locally circulating, such as the recent finding on the circulation of DENV2 in Kassala state in 2018 [18]. The aim of this study is to conduct a systematic review and meta-analysis to better estimate the true burden of DENV in Sudan.

## 2. Materials and Methods 

### 2.1. Searching Strategy

The literature review and records extraction were done by searching several databases including PubMed, Medline, EMBASE, Web of Sciences, Scopus, ScienceDirect, and Google Scholar using the following combination of search terms: (“Sudan”) and (“dengue” or “dengue fever” or “dengue prevalence” or “dengue incidence” or “dengue virus” or “severe dengue” or “DENG” or “DENV” or “الضنك (Arabic word for dengue)”) and the last search was October 2019. Literature was extracted independently by two authors.

### 2.2. Inclusion Criteria

Studies were retrieved regardless of region, year in which the survey was implemented, or study design. We only included reports from Sudan. We included cohort studies, case series, or case controls that provided data on dengue exposure. Enzyme linked immunosorbent assays (ELISAs) for IgG and/or IgM were the main laboratory tests to diagnose DENV infection, and a few IgG-based studies included additional PCR testing for infection confirmation and serotype identification. Articles were excluded that did not report study design and/or dengue testing results.

### 2.3. Data Extraction and Validity Assessment 

The New Castle-Ottawa Scale (NOS) guideline was followed for assessing the non-randomization and the risk of bias for the extracted studies in the meta-analysis [19]. From each eligible study we extracted the following information: author, location, year of the survey, methodology, study design, sample size, type of diagnostic testing, results of dengue testing, and demography of the research participants. 

### 2.4. Statistical Analysis

STATA-13 (Stata Corp., College Station, TX, USA) was used for the data analysis. Random effects modeling was used to analyze the extracted data. The primary outcome measure was DENV seroprevalence. The standard error of the prevalence estimation was calculated using binomial probability distribution. Overall and subgroup pooled effect size was estimated using a random effects model by calculating the pooled proportion and confidence interval [20]. The heterogeneity of different studies was assessed by calculating the value of the chi-square (Q) at 10% significant level. Heterogeneity across the results of included studies was assessed by I2, while tau-square (Tue2) was used to assess the between-study heterogeneity [21]. We used the Preferred Reporting Items for Systematic Review and Meta-Analysis (PRISMA) guidelines of reporting [22].

Since the studies were conducted by different researchers in different settings, the similarity between studies is very unlikely. This is why we performed our analysis following the random effect model, in which we assumed that true effect size varies from one study to another, which is different from a fixed-effect model as the random effect model estimates the mean of a distribution of effects. In our analysis model, to avoid analytical bias both variances, within-studies variance and between-studies variance, were counted for in the analysis. This model depends on the weight assigned to each study. So, the goal here is to estimate the mean effect in a range of studies, and the overall estimate not to be affected by large or small studies.

## 3. Results

**Figure 1:**
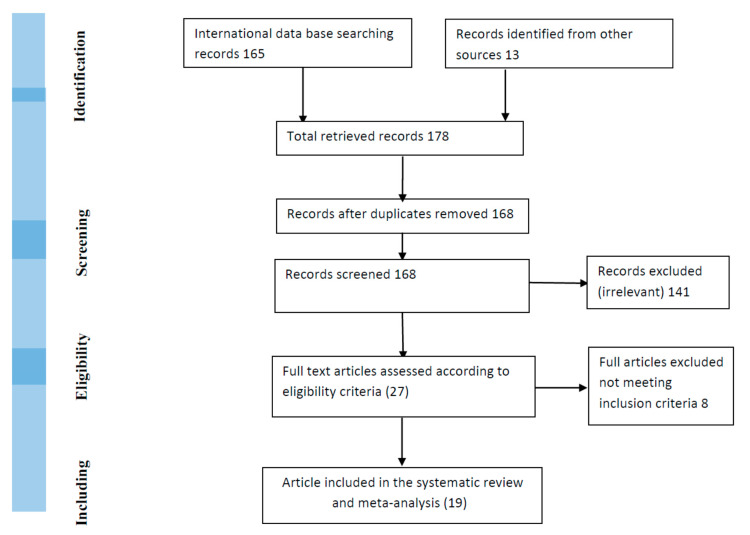
Study inclusion flow chart.

Of the 178 retrieved studies, 10 duplicates were excluded, and from the 168 identified remaining records, 141 were excluded because they were reporting non-dengue infections but citing dengue reports. The twenty-seven studies were screened, and additional 8 records were excluded due to lack of data on research participants and infection rates (Figure 1).

Thus, nineteen studies fully satisfied the inclusion criteria and were included in the analysis. The selected studies covered multiple regions of Sudan: thirteen were reported from the Eastern region of the country, three were from the West, two were from Central Sudan, and a single study was from North Sudan (Table 1). 

**Figure 2:**
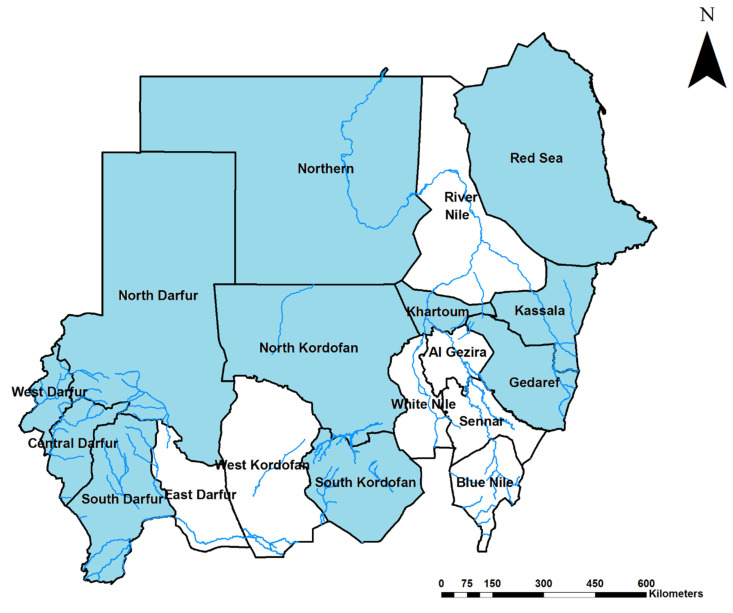
Map of Sudan areas highlighted in light blue indicate states where dengue virus infection has been reported.

Thirteen studies were cross-sectional hospital-based, five were cross-sectional community-based, and a single study was a hospital-based retrospective cohort study. Most of these studies were implemented either during or following recognized disease epidemics. Dengue infections were documented in 11 out of 18 Sudanese states (Figure 2), indicating that 65.1% of the country’s total population is at risk of dengue infection.

**Figure 3:**
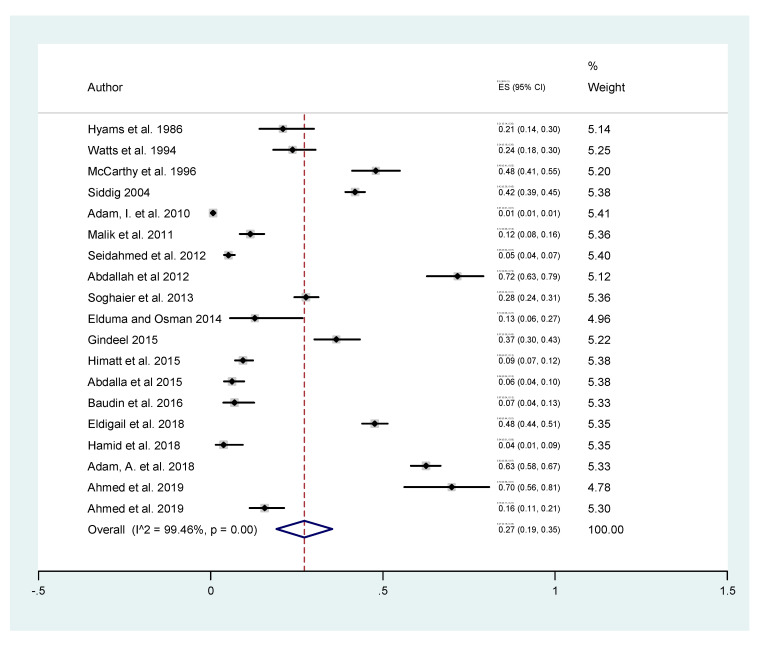
The estimated burden of dengue based on the analysis of all included studies.

The meta-analysis showed that the overall seroprevalence of DENV in Sudan is 27% (95% C.I. 19–35%). The analysis highlighted the variation in DENV seroprevalence, which ranged between 1% and 72%. This variation in DENV estimates (99.46%) and *p* = 0.00 could be attributed to differences in factors related to disease transmission/infection rate including the spatiotemporal variation in addition to the study design and the diagnostic tool used to investigate DENV infection (Figure 3).

**Figure 4:**
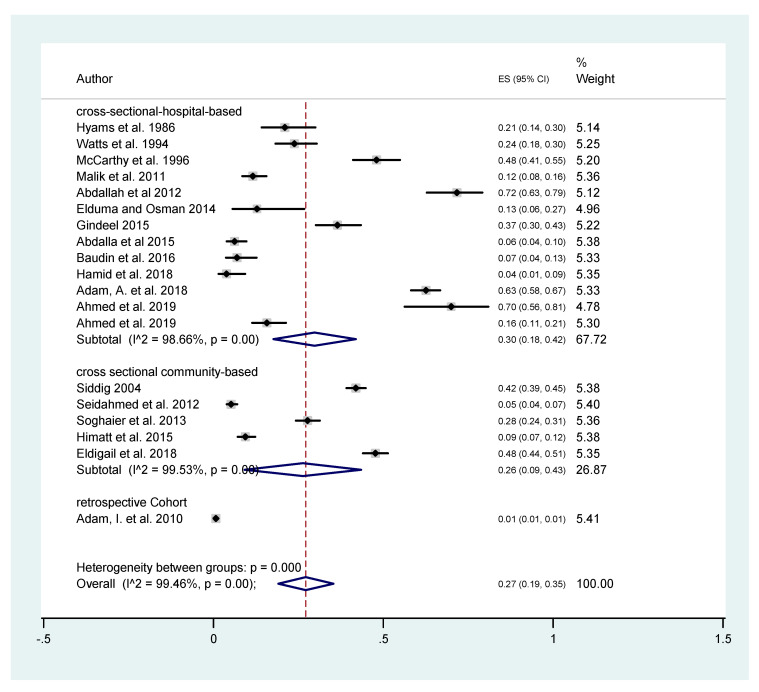
The estimated burden of dengue based on sub-group analysis according to study design.

To more accurately estimate the DENV seroprevalence in Sudan, the included studies were sub-grouped and analyzed according to the type of study (Figure 4). The studies were classified into two groups: hospital-based cross-sectional studies (*n* = 13) and community-based cross-sectional studies (*n* = 5). This analysis was performed to compare the seroprevalence among patients presented in healthcare facilities and the public community. In the hospital-based group, the estimated DENV seroprevalence was 30% (95% C.I. 18–42%) and the I2 was 98.66%. The estimated seroprevalence of DENV infections from the community-based studies was 26% (95% C.I. 9–43%) and the I2 was 99.53%.

**Figure 5:**
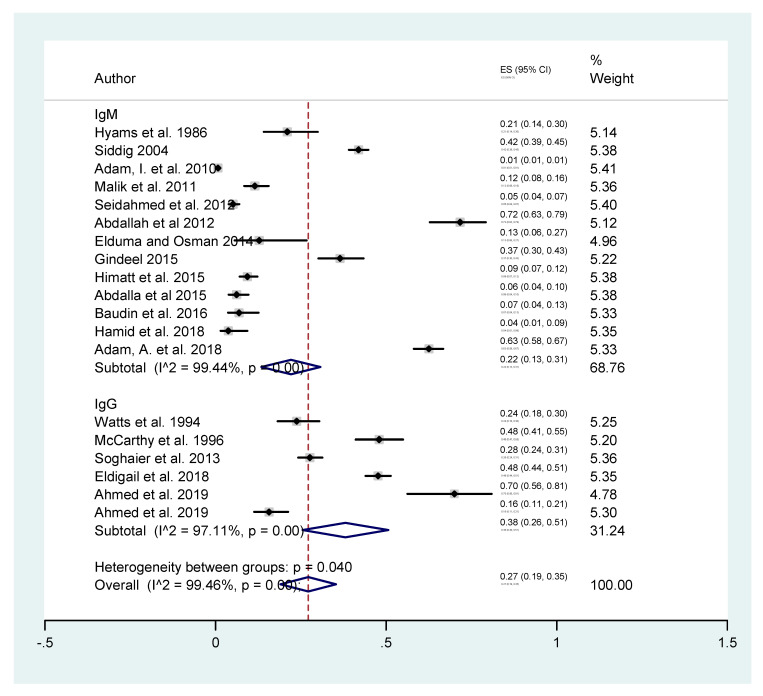
The estimated burden of dengue based on sub-group analysis according to diagnostic test.

Further analysis was done by sub-grouping and analyzing the studies according to the laboratory test that was used to diagnose previous exposure to DENV (IgG) and recent acute infection (IgM) (Figure 5). The estimated seroprevalence of DENV as measured by IgM ELISA was 22% (95% C.I. 13–31%) and the I2 was 99.44%. The estimated seroprevalence of DENV as measured by IgG ELISA was 38% (95% C.I. 26–51%) and the I2 was 97.11%, consistent with the more durable nature of the IgG antibody response.

**Figure 6:**
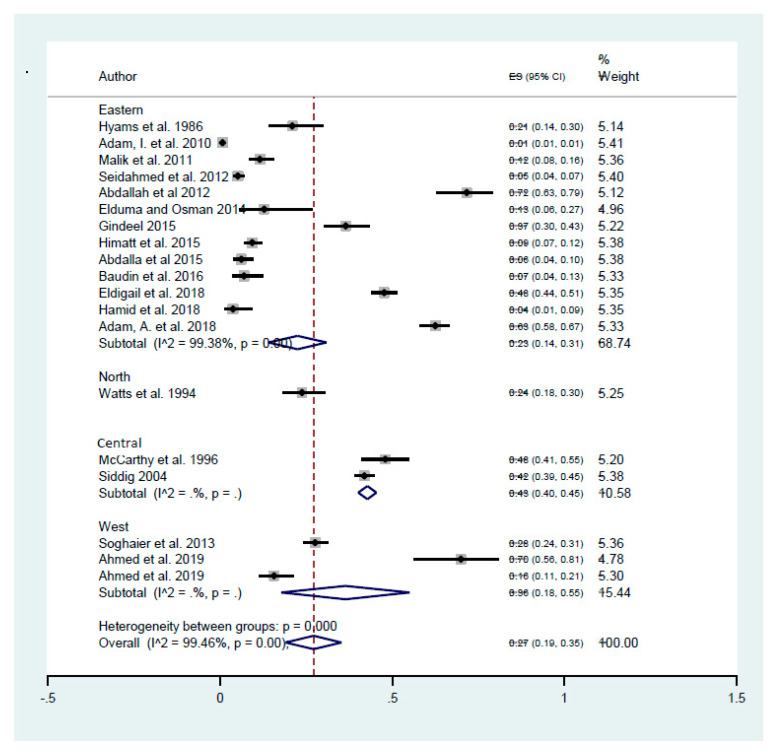
The estimated burden of dengue based on sub-group analysis according to geographical location of the study (regions).

Additionally, the regional prevalence of the disease was estimated by sub-group analysis according to the geographical location of the studies site: North, East, South, Central, or Western Sudan (Figure 6). The estimated seroprevalence of DENV infections was 23% (95% C.I. 14–31%) in East Sudan, 43% (95% C.I. 40–45%) in Central Sudan, 24% (95% C.I. 18–30%) in North Sudan, and 36% (95% C.I. 18–55%) in Western Sudan.

Additional statistical tests were performed to investigate the heterogeneity and significance of the overall DENV seroprevalence, the seroprevalence per study types, locations, and the diagnostic tools used for the detection of DENV. The analysis showed that 99.46% of the seroprevalence estimates is attributed to heterogeneity in the analysis with high significance (*p* = 0.00), which limits the effect of any confounding factors over the results (Tables S1–S7).

## 4. Discussion

This is the first systematic review and meta-analysis investigation on DENV seroprevalence in Sudan. The results of the meta-analysis revealed that the overall seroprevalence of DENV in Sudan was 27%, and that DENV circulates in many regions across the country. Nevertheless, this study highlighted that the lack of a sustainable national surveillance system in Sudan for DENV and other arboviral infections has left the population at substantial risk. Surveys in some cases were separated by as much as 21 years. A more thorough and consistent approach to arbovirus surveillance is urgently needed to identify epidemics and direct precious public health resources accordingly [14,17]. A proper communication channel needs to be integrated in this surveillance system to ensure the timely sharing of disease prevalence and epidemics [35].

Sub-group analysis was performed to establish more accurate estimates of the disease prevalence and exclude the potential bias introduced by combining different studies together. This includes analyzing the studies sub-grouped by study type, location, and the test performed to detect DENV infection. Temporal variation was investigated by sub-grouping studies by the diagnostic test performed, IgG to assess the previous exposure to the virus, and IgM to assess the recent acute infection [33]. The prevalence of the DENV-IgG 38% (95% C.I. 26–51%) was significantly higher than DENV-IgM 22% (95% C.I. 13–31%) indicating that the DENV transmission was likely both endemic as well as epidemic [33]. The estimated seroprevalence of DENV was similar between community-based and hospital-based studies with 26% (95% C.I. 9–43%) and 30% (95% C.I. 18–42%), respectively, which suggests that DENV is endemic in throughout the country. The spatial variation of the DENV seroprevalence was investigated by sub-grouping studies by the regions of the country where the studies were implemented. The analysis showed that DENV seroprevalence was highest in Central (43%) and Western (36%) Sudan, followed by similar seroprevalence in Northern (24%) and Eastern (23%) regions of the country. Similar regional variation and heterogeneity of DENV prevalence are reported in the neighbor countries including Kenya, where the prevalence rates range between 34.17% in the coastal areas and 1.9% in Western regions [36,37,38], as well as in Saudi Arabia where it ranges between from 0.1% to 31% [39]. Dengue epidemics also occurred in Egypt in 1927 and affected the whole country [40]. Furthermore, a large increase (243%) of DENV infections was reported in Eritrea between 2005 and 2014 [41].

Our findings showed almost similar increase in infection rates from 0.7% to 72% within one year 2009/2010 [25,27], which highlights that the prevalence of DENV is remarkably increasing over time with recent studies reporting prevalence higher than 70% [14]. Alternatively, this significant change in prevalence estimates could be attributed to improvement in testing and reporting, as well as the time of samples collection whether before, during, and after epidemics. The latter explanation is more likely to be the case because of the lack of timely sharing of health emergency information in the country [35]. Additionally, most of the Sudanese population is internally displaced persons and this high population dynamic due to natural and man-made disasters including armed-conflicts, famine, flooding, and other health emergencies could explain discrepancies between the IgM and IgG seroprevalence estimates because of the local population turnover [13,14,17].

Moreover, the geographical distribution of DENV in Sudan is continuously expanding; by 2012 DENV emerged in West Sudan and between 2012 to 2017 DENV infections were reported from seven states for the first time [13,14,33,34]. Alarmingly, DENV seems to be prevalent in all Sudan borders with neighboring countries (Figure 2), including Eritrea, Ethiopia, South Sudan, Central African Republic, Chad, Libya, Egypt, and Saudi Arabia across the Red Sea, which imposes additional global health risk of cross-border transmission, particularly with the open borders and free mobility between these countries.

The need for an arboviral disease surveillance program in Sudan is crucial and urgent to investigate the actual prevalence and burden of arboviruses including DENV, vector distribution, the serotypes and genotype of relevant viruses. It will also help accurately depict the risk and dynamics of DENV and to inform policies on disease prevention and control. Additionally, it will direct the vector control efforts and identify the risk factors associated with disease transmission in the varied regions of Sudan. Such studies will generate information crucial for the development of effective public health policy for the prevention and control of dengue infections, and can provide invaluable insight into arbovirus research [17].

This review could have been much richer in data and information and could have offered more in-depth insights about DENV transmission in Sudan. However, these limitations are due to the lack of local and international support for arboviral diseases research and control activities in Sudan, as well as the lack of sharing live data and information about health emergencies by the health authorities [35]. Therefore, here we urge international donors, research and diseases control funding agencies, and international health partners and research institutes to support and collaborate with institutions in endemic countries to investigate, prevent, and control endemic diseases through funding, capacity building, and partnership.

## 5. Conclusions

In conclusion, this study highlights the high burden, up to 43%, and rapid expansion of dengue virus distribution in Sudan, and the growing need for a systemic investigation of dengue burden in Sudan. Dengue infection has been reported in different areas of Sudan and should be considered in the differential diagnosis for any fever case in Sudan. Surveillance and prevention measures are needed to curb the impact of this rapidly growing public health threat. Particularly, this year, with the current interruption of vector control activities and disease surveillance due to the COVID-19 pandemic, unless alternative measures are taken then devastating epidemics of DENV are inevitable country-wide. However, considering that the main vector of DENV, *Aedes aegypti*, breeds mainly in man-made water containers, and these days most of the population is staying home due to the national lockdown, everyone could contribute in controlling the vector in their homes by limiting breeding sites. Here we call for such a national initiative which would decrease the risk of several mosquito-borne diseases in Sudan, including DENV.

## Figures and Tables

**Table 1:** List of studies that met the criteria to be included in the analysis.

No	Year of Survey	Region	State	Study Type	Diagnostic Test	Sample Size	No of Cases	Reported Prevalence	Detected Serotype	Mean Age	Sex	Reference
1	1984	East	Red Sea	Cross-sectional hospital-based	ELISA-IgM	100	21	21%	DENV1 and 2	-	M + F	[23]
2	1988	Central	Khartoum	Cross-sectional hospital-based	ELISA-IgG	196	97	33%	DENV2	20	M + F	[11]
3	1989	North	Northern	Cross-sectional hospital-based	ELISA-IgG	185	44	24%	DENV2	34	M + F	[12]
4	2000	Central	Khartoum	Cross-sectional community-based	ELISA-IgM	1157	485	42%	-	-	M + F	[24]
5	2005	East	Red Sea	Cross-sectional hospital-based	ELISA-IgM	312	36	12%	DENV3	5–15	M + F	[10]
6	2009	East	Red Sea	Retrospective Cohort	ELISA-IgM	10,820	78	1%	-	-	Pregnant women	[25]
7	2009	East	Red Sea	Cross-sectional community-based	ELISA-IgM	791	41	5.2%	-	30	M + F	[26]
8	2010	East	Kassala	Cross-sectional hospital-based	ELISA-IgM	113	81	72%	-	25.5	M + F	[27]
9	2010	East	Red Sea	Cross-sectional hospital-based	ELISA-IgM	200	73	37%	DENV2–4	25	M + F	[9]
10	2011	East	Kassala	Cross-sectional community-based	ELISA-IgM	491	46	9%	-	40	M + F	[28]
11	2012	East	Kassala	Cross-sectional hospital-based	ELISA-IgM	275	17	6%	-	17.8	M + F	[29]
12	2012	East	Red Sea	Cross-sectional hospital-based	ELISA-IgM	130	9	7%	-	-	F	[30]
13	2012	East	Red Sea	Cross-sectional hospital-based	ELISA-IgM	39	5	13%	-	26	Pregnant women	[31]
14	2012	West	South Kordofan	Cross-sectional community-based	ELISA-IgG	615	170	28%	-	37.5	M + F	[32]
15	2013	East and West	Red Sea, Kassala, North Kordofan	Cross-sectional hospital-based	ELISA-IgM	483	302	63%	DENV1–4	40.7	M + F	[33]
16	2015	West	North Darfur	Cross-sectional hospital-based	ELISA-IgG	50	35	70%	DENV1 and 3	37.8	M + F	[14]
17	2016	West	Central, North, South, and West Darfur	Cross-sectional hospital-based	ELISA-IgM	204	32	16%	DENV1 and 3	-	M + F	[13]
18	2017	East	Gadaref	Cross-sectional community-based	ELISA-IgG	701	334	48%	-	-	M + F	[34]
19	2017	East	Kassala	Cross-sectional hospital-based	RT-PCR	106	4	4%	DENV2	-	-	[18]

## Supplementary Materials

The following are available online at https://www.mdpi.com/2414-6366/5/3/120/s1. Table S1: Heterogeneity test for the general analysis of all included dengue reports. Table S2: Heterogeneity tests for the sub-group analysis by study type. Table S3: Significance tests of estimates for the sub-group analysis by study type. Table S4: Heterogeneity tests for the sub-group analysis according to diagnostic test. Table S5: Significance tests of estimates for the sub-group analysis according to diagnostic test. Table S6: Heterogeneity tests for the sub-group analysis according to Study location. Table S7: Significance tests of estimates for the sub-group analysis according to study location.
