# Supplementary material for: High Seroprevalence of Dengue Virus Infection in Sudan: Systematic Review and Meta-Analysis
Source: Trop Med Infect Dis. 2020 Jul 18;5(3):120. doi: 10.3390/tropicalmed5030120 (PMC7559303; doi:10.3390/tropicalmed5030120)
Supplement: Supplementary file 1 [file tropicalmed-05-00120-s001.pdf]

Supplementary Materials:

Table S1: Heterogeneity Test for the general analysis of all included dengue reports.

**Statistical Test**	**Results**	**Degree of Freedom**	**Significant (P value)**
Heterogeneity chi^2	3315.42	18	0.00
I^2 (variation in ES attributable to heterogeneity)	99.46%	-	-
Estimate of between-study variance Tau^2	0.03		
Test of ES	0		
Z	6.57		0.00

Table S2: Heterogeneity Tests for the sub-group analysis by study type.

**Heterogeneity degrees**	**Results**	**Degree of Freedom**	**Significant (P value)**	**I^2****
Cross-sectional hospital-based	895.72	12	0.00	98.66%
Cross-sectional community-based	852.23	4	0.00	99.53%
Retrospective Cohort	0	-	-	-
Overall	3315.42	18	0.00	99.46%
Test for heterogeneity between sub-groups	30.49	2	0.00	-

** I^2: the variation in ES attributable to heterogeneity)

Table S3: Significance tests of Estimates for the sub-group analysis by study type.

**Significance test**	**Z**	**Significant (P value)**
Cross-sectional hospital-based	4.80	0.00
Cross-sectional community-based	3.01	0.00
Retrospective Cohort	8.86	0.00
Overall	6.57	0.00

Table S4: Heterogeneity Tests for the sub-group analysis according to diagnostic test.

**Heterogeneity degrees**	**Results**	**Degree of Freedom**	**Significant (P value)**	**I^2****
IgM	2126.24	12	0.00	99.44%
IgG	173.30	5	0.00	97.11%
Overall	3315.42	17	0.00	99.46%
Test for heterogeneity between sub-groups	4.24	1	0.04	-

** I^2: the variation in ES attributable to heterogeneity)

Table S5: Significance tests of Estimates for the sub-group analysis according to diagnostic test..

**Significance test**	**Z**	**Significant (P value)**
IgM	5.01	0.00
IgG	5.95	0.00
Overall	6.57	0.00

Table S6: Heterogeneity Tests for the sub-group analysis according to Study location.

**Heterogeneity degrees**	**Results**	**Degree of Freedom**	**Significant (P value)**	**I^2****
East	1943.23	12	0.00	99.38%
North	-	0	-	
Central	-	1	-	
West	-	2	-	
Overall	3315.42	18	0.00	99.46%
Test for heterogeneity between sub-groups	46.00	3	0.00	-

** I^2: the variation in ES attributable to heterogeneity)

Table S7: Significance tests of Estimates for the sub-group analysis according to Study location.

**Significance test**	**Z**	**Significant (P value)**
East	5.22	0.00
North	7.60	0.00
Central	31.83	0.00
West	3.83	0.00
Overall	6.57	0.00
